# A Porous Nano-Micro-Composite as a High-Performance Bi-Functional Air Electrode with Remarkable Stability for Rechargeable Zinc–Air Batteries

**DOI:** 10.1007/s40820-020-00468-4

**Published:** 2020-06-17

**Authors:** Yasir Arafat, Muhammad Rizwan Azhar, Yijun Zhong, Xiaomin Xu, Moses O. Tadé, Zongping Shao

**Affiliations:** 1 WA School of Mines: Minerals, Energy and Chemical Engineering (WASM-MECE), Curtin University, Perth, WA 6845 Australia; 2 School of Engineering, Edith Cowan University, Perth, WA 6027 Australia; 3 State Key Laboratory of Materials-Oriented Chemical Engineering, College of Chemical Engineering, Nanjing Tech University, Nanjing, 210009 Jiangsu People’s Republic of China

**Keywords:** BSCF perovskites, ZIF-67, Porous carbon, Zn–air batteries, Oxygen evolution reaction, Oxygen reduction reaction

## Abstract

The development of bi-functional electrocatalyst with high catalytic activity and stable performance for both oxygen evolution/reduction reactions (OER/ORR) in aqueous alkaline solution is key to realize practical application of zinc–air batteries (ZABs). In this study, we reported a new porous nano-micro-composite as a bi-functional electrocatalyst for ZABs, devised by the in situ growth of metal–organic framework (MOF) nanocrystals onto the micrometer-sized Ba_0.5_Sr_0.5_Co_0.8_Fe_0.2_O_3_ (BSCF) perovskite oxide. Upon carbonization, MOF was converted to porous nitrogen-doped carbon nanocages and ultrafine cobalt oxides and CoN_4_ nanoparticles dispersing inside the carbon nanocages, which further anchored on the surface of BSCF oxide. We homogeneously dispersed BSCF perovskite particles in the surfactant; subsequently, ZIF-67 nanocrystals were grown onto the BSCF particles. In this way, leaching of metallic or organic species in MOFs and the aggregation of BSCF were effectively suppressed, thus maximizing the number of active sites for improving OER. The BSCF in turn acted as catalyst to promote the graphitization of carbon during pyrolysis, as well as to optimize the transition metal-to-carbon ratio, thus enhancing the ORR catalytic activity. A ZAB fabricated from such air electrode showed outstanding performance with a potential gap of only 0.83 V at 5 mA cm^−2^ for OER/ORR. Notably, no obvious performance degradation was observed for the continuous charge–discharge operation for 1800 cycles over an extended period of 300 h.

**Electronic supplementary material:**

The online version of this article (10.1007/s40820-020-00468-4) contains supplementary material, which is available to authorized users.

## Highlights

- A novel high-performance bi-functional electrocatalyst was fabricated by in situ growth of ZIF-67 polyhedrons onto Ba_0.5_Sr_0.5_Co_0.8_Fe_0.2_O_3_ (BSCF) particles.
- Strong coupling between different phases endowed the robust structure to the composite, which translated into stable charging/discharging operation for 300 h. And the new interfacial phases significantly promoted the catalytic activity, stability and electrical conductivity.

**Electronic supplementary material:**

The online version of this article (10.1007/s40820-020-00468-4) contains supplementary material, which is available to authorized users.

## Graphical abstract

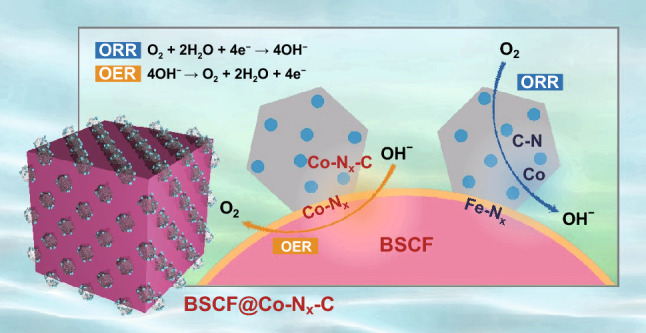

## Introduction

Quick global population growth and fast increase in world economy have led to the huge consumption of fossil fuels, while the scarcity of fossil fuels and growing environmental legislations have aroused the quest for alternative energy resources that should be renewable and clean [1]. Recently, sustainable energies such as hydro, wind and solar have drawn a great deal of interest by virtue of their eco-friendliness, economical attractiveness and ubiquitous availability [2]. Regardless of these benefits, these energies are intermittent; thereby, it is crucial to locate some viable energy storage devices. In this context, rechargeable batteries are considered as a source of storing harvested energy from the given resources [3, 4].

Among the various types of electrochemical energy storage devices, Zn–air batteries (ZABs) have received particular attention because of their environmental friendliness, cost effectiveness, high energy density (470 Wh kg^−1^) and abundance in raw materials. However, their placement on commercial scale is obstructed by the sluggish kinetics related to the oxygen reduction reaction (ORR) while discharging and oxygen evolution reaction (OER) in the course of charging, which eventually reduces the rate performance as well as round-trip efficiency. Consequently, the development of efficient air electrode, which may exhibit high and stable activity for both ORR and OER, is vital to realize high-performance Zn–air batteries [5].

Considering their abundance, low cost and environmental benignity, transition metal compounds have emerged as potential noble metal-free electrocatalysts for various chemical reactions, including ORR and OER. For example, cobalt oxides (CoO/Co_3_O_4_) [6, 7], Co_4_N and Co-based perovskites [8–11] are known to exhibit high intrinsic catalytic activity for OER in alkaline media. However, their apparent activity may be limited by the poor electronic conductivity (cobalt oxides and perovskite oxides) and low surface area (perovskite oxides), and their durability may be limited by the high tendency of sintering/aggregation. These challenges may be resolved by anchoring transition metal oxide/nitride nanoparticles into porous carbon network. These carbon materials provide sufficient electronic conductivity to enhance charge transfer on the one hand and greatly increase the dispersion of transition metal oxides so as to increase the number of active sites and to suppress the sintering/aggregation of the nanoparticles on the other hand, improved catalytic activity and durability are then expected [12]. Furthermore, the doping of N heteroatom in the carbon lattice enhances the ORR activity and stability. Via this compositing strategy, bi-functional electrocatalyst with high activity and durability for both ORR and OER is expected.

Metal organic frameworks (MOFs), wherein transition metals are coordinated by organic ligands in a three-dimensional network [13–17], are excellent precursors for the synthesis of porous carbon-/nitrogen-doped carbon architecture, as well as metallic nanoparticle-modified porous carbon materials. For example, zeolitic imidazole framework-67 (ZIF-67) has been extensively employed for the synthesis of porous Co_4_N/Co–N_x_–C composite. Upon carbonization under N_2_ atmosphere at elevated temperature, Co–N_x_–C nanocomposite, which partially inherited the porous framework of MOFs, was formed. Such material demonstrated high catalytic activity for ORR [15]. By partial oxidation of Co–N_x_–C with a successive thermal treatment in air with the formation of Co/CoO_x_–N–C composite leads to good OER activity as well [12, 16]. However, during the oxidation of cobalt in Co–N_x_–C composite, the partial oxidation of carbon may also happen, which will impair the conductivity and continuity of the carbon framework. Another major concern in applying MOFs in alkaline solution is the leaching of metallic and/or organic moieties into the reaction medium from the MOFs [18]. In addition, due to the fixed ratio of metal ion and carbon in the MOFs, there is small room to tailor the molar ratio of metal ion to carbon in the carbonized sample, while such ratio is closely related to the catalytic OER/ORR performance. Therefore, the incorporation of external metal oxides into MOFs may further improve the ORR/OER performance through optimizing the metal oxides to carbon ratio in the composite. Moreover, when a synergistic effect is developed between the external metal oxides and the porous carbon as derived from MOFs, a further improvement in the ORR/OER activity may be experienced, while intimate contact of them is a key for the creation of such synergy and for achieving the stable activity. Therefore, the development of a suitable synthesis method that allows the thorough dispersion of metal oxides, inside the carbon framework as derived from MOFs and suppressing the leaching of metallic or organic species by creating strong interaction between the metal oxide and the carbon is highly wanted.

Herein, we proposed a novel porous nano-micro-composite, derived from BSCF micrometer-sized particles and ZIF-67 MOF, as an ideal bi-functional electrocatalyst for ZABs with high conductivity, superior activity and impressive durability. We developed a novel strategy that allows the in situ growing of ZIF-67 nanocrystals on the surface of BSCF oxide. Upon carbonization, ZIF-67 nanocrystals transformed into cobalt/cobalt oxides and Co_4_N and N-doped porous carbon nanocages, which tightly anchored and covered the BSCF particles. In this way, BSCF particles were protected against sintering to allow thorough dispersion and large number of active sites. In situ growth of ZIF-67 crystals onto BSCF surface provided intimate contact, which offered the inhibition of leaching of metallic and/or organic species, good electrical conductivity and structural stability. Furthermore, their strong interaction promoted the synergy between different components, while BSCF functioned as a catalyst for stimulating the graphitization during carbonization of MOFs and provided additional source of cobalt/iron for tailoring the ratio of transition metal to carbon for optimizing electrocatalytic activity. As a result, superior bi-functional activity and remarkable stability of the porous nano-micro-composite were realized, which eventually demonstrated outstanding performance in ZABs. Such a strategy is also suitable for the preparation of other transition metal oxides MOFs-based dual functional catalysts, thus providing a new way for the performance enhancement of ZABs.

## Experimental

### Catalysts Synthesis

#### Synthesis of BSCF

The synthesis of BSCF Perovskite oxide was conducted by employing ethylene diamine tetra acetic acid–citric acid (EDTA-CA) complexing sol–gel method [19]. Briefly, corresponding stoichiometric amounts of Ba(NO_3_)_2_, Sr(NO_3_)_2_, Co(NO_3_)_3_·6H_2_O, and Fe(NO_3_)_3_·9H_2_O were dissolved in ultrapure water and EDTA and CA (complexing agent) with a molar ratio of 1:2:2, respectively, added together, followed by the addition of ammonia water for the purpose of adjusting pH. The obtained solution was heated at about 100 °C while stirring until a clear viscous solution was obtained. Subsequently, the obtained viscous solution was heated at 250 °C for 5 h in the furnace to get a black solid precursor. Eventually, the calcination of precursor was conducted at 1100 °C for the duration of 5 h in the atmosphere of air and finally BSCF powder obtained after grinding.

#### In Situ Growth of ZIF-67 Crystals Over BSCF Surface

For in situ growth of ZIF-67 crystals over BSCF surface, firstly, pluronic F-127 (surfactant) dissolved in methanol solvent, and then, as-prepared BSCF oxide powder was evenly dispersed in this solution by employing continuous magnetic stirring for 1 h. Subsequently, salt of cobalt nitrate was added in the same solution. Also, 2-methylimidazole was dissolved separately in methanol until a clear solution was achieved. Later, solution was added in the former one while stirring was in progress at room temperature. After 2 h, mixing was stopped and reaction mixture aged for 24 h and dried at room temperature. By employing the same synthesis strategy, four different BSCF/ZIF composites were prepared. The concentration of ZIF-67 precursors (cobalt nitrate and 2-methyl imidazole) was kept as fixed; however, the concentration of BSCF powder was varying (0.032, 0.062, 0.093 and 0.124 g), and the samples were named as BCZ1, BCZ2, BCZ3 and BCZ4, respectively.

#### Synthesis of ZIF-67

For the purpose of comparison, ZIF-67 rhombic dodecahedrons were fabricated as per the previous reports with minor modifications [20]. In brief, the corresponding amount of cobalt nitrate salt and pluronic F-127 (surfactant) was dissolved in methanol solvent. On the other hand, another solution based on 2-methylimidazole and methanol was separately prepared. Formerly, prepared pink solution was dissolved in the clear solution, while stirring at room temperature. Subsequent to the proper mixing, stirring was stopped; precipitation of ZIF-67 occurred. The reaction mixture aged for 24 h and finally dried at room temperature.

The carbonization of as-synthesized (BCZ1, BCZ2, BCZ3 and BCZ4) composites and ZIF-67 was conducted in two steps in the atmosphere of N_2_ in the tube furnace. Initially, the temperature was increased from room temperature to 350 °C at 1 °C min^−1^ (ramping rate) and kept at the final temperature for 1 h. Subsequently, the temperature is raised form 350 °C till 750 °C at the ramp of 2 °C min^−1^ and retained at this temperature for 2 h. After carbonization, ZIF-67 was named as C-ZIF-67.

#### Synthesis of the Physical Mixture of C-ZIF-67 and BSCF

For the purpose of comparison, the physical mixture of BSCF and carbonized ZIF-67 was prepared by conducting physical mixing through ultrasonication process using different ratios of BSCF to C-ZIF-67 (20:80, 30:70, 40:60, 50:50 and 0:100).

### Characterization of Catalysts

Phase analysis of samples was conducted by employing X-ray diffraction (XRD), and signals were recorded in the range 10–90° (2*θ*), on Advance X-ray diffractometer, utilizing Cu Kα radiation having the voltage of 40 kV as well as 40 mA current. The morphology of carbonized samples was studied by scanning electron microscopy (SEM) analysis (Zeiss NEON 40 EsB CrossBeam-JEOL S4800). The high-resolution transmission electron microscopy (HRTEM) TEM and STEM–HAADF micrographs together with elemental mapping were secured on JEOL instrument (JEM-2100) using an electron source (200 kV). Brunauer–Emmett–Teller (BET) surface area and pore dimensions were recorded by N_2_ (adsorption/desorption) isotherms by employing Micromeritics TriStar II instrument. X-ray photoelectron spectra (XPS) signals were monitored with Al Kα X-ray, employing a Kratos AXIS Ultra DLD system under UHV conditions. The analysis of the data was carried out by CasaXPS software. The carbon content of samples was measured by thermogravimetric analysis employing STAR^e^ system, Mettler Toledo instrument after heating at 700 °C in the atmosphere of air.

### Electrochemical Measurements

The electrochemical measurements were carried out in a three-electrode glass cell consisting of Ag/AgCl in 4 M KCl solution (reference electrode), platinum wire (counterelectrode) and a glassy carbon of 5 mm diameter (working electrode). An aqueous solution of 0.1MKOH (pH ≈ 12.8) was utilized as an electrolyte, saturated with O_2_ to conform the O_2_/H_2_O equilibrium. Linear sweep voltammograms (LSVs) were acquired employing RDE at 1600 rpm at a scan rate of 5 mV s^−1^. All of the data obtained through electrochemical measurements were recorded on a CHI 760E workstation (bipotentiostat, CH Instruments, Inc., USA). Potentials recorded from the Ag/AgCl, reference electrode were converted to reversible hydrogen electrode potentials (*E*_RHE_) by using Eq. 1:

1 $$E_{\text{RHE}} = E_{\text{Ag/AgCl}} + 0.199 + 0.0591 \times {\text{pH}}$$

The catalyst ink was prepared using 12 mg of electrocatalyst (In case of BSCF, 10 mg of BSCF + 2 mg of carbon black/super P) by making dispersion in a solution (900 μL of absolute ethanol and 100 μL of Nafion) under sonication for the period of 1 h. Subsequently, 5 μL of resultant suspension was drop cast on glassy carbon electrode, eventually to get the catalyst loading of 0.255 mg cm_disk_^−2^ after drying overnight in the air.

During ORR, the number of transferred electrons and the release of H_2_O_2_ (%) were monitored by the rotating ring-disk electrode (RRDE) tests, consisting of glassy carbon electrode (5.61 mm) surrounded by Pt ring (outer diameter = 7.92 mm; inner diameter = 6.25 mm). During the test, ring potential was retained at 1.21 V (vs. RHE) to quantify the generation of H_2_O_2_ species. The number of transferred electrons (*n*) as well as H_2_O_2_ released (%H_2_O_2_) during the course of ORR was computed using Eqs. 2 and 3:

2 $$n = 4I_{\text{d}}/\left({I_{\text{d}} + \frac{{I_{\text{r}}}}{N}} \right)$$

3 $$\% {\text{H}}_{2} {\text{O}}_{2 } = 200\frac{{I_{\text{r}}}}{N}/\left({I_{\text{d}} + \frac{{I_{\text{r}}}}{N}} \right)$$

where *I*_d_ and *I*_r_ stand for the ring current and disk currents, respectively.

### Assembling and Testing of Zn–air Battery

The assembly of liquid Zn–air battery consists of air cathode, based on a carbon paper (consist of two sides, water-facing side and air diffusion layer on air-facing side), an electrolyte (6 M KOH + 0.2 M Zn(Ac)_2_) and a polished zinc plate as anode. The catalyst ink was prepared by using 10 mg of carbonized samples without adding any additional conductive material. The dispersion of material is made in a solution (1000 μL of absolute ethanol and 100 μL of Nafion) under sonication for a period of 1 h. Subsequently, 55 μL of resultant suspension was drop cast on water-facing, hydrophobic carbon paper (0.4 cm in diameter), eventually to get the catalyst loading of 1 mg cm_paper_^−2^ after drying overnight in the air. The appraisal of battery performance was conducted by employing a CHI 760E, potentiostat (CH Instrument Co.) instrument and LAND testing system.

## Results and Discussion

### Physicochemical Characterizations of Catalysts

**Scheme 1:**
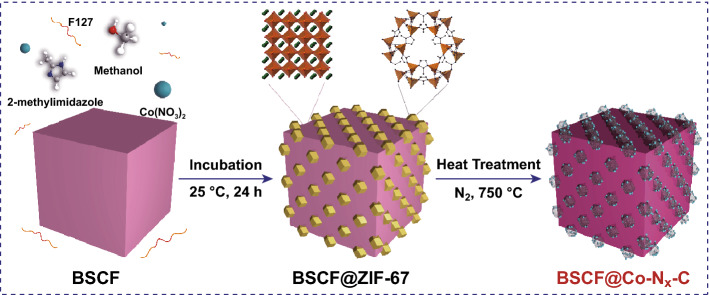
A schematic description of in situ growth of ZIF-67 crystals on BSCF surface and carbonization of prepared sample

Scheme 1 illustrates the preparation sequence of the BSCF@Co–N_x_–C electrocatalysts. We prepared BSCF perovskite particles with their surface anchored with rich nanocages of zeolite imidazolate frameworks (ZIF-67) through in situ growth of ZIF-67 obtained from the solution of cobalt nitrate and 2-methylimidazole in the presence of BSCF particles. To realize this growth, micrometer-sized BSCF oxide powder was first synthesized based on EDTA–citrate complexing sol–gel method followed by pyrolysis and then homogenously dispersed in a pluronic F-127 solution as a surfactant through continuous magnetic stirring. Subsequently, the growth of ZIF-67 nanocubes over the surface of BSCF substrate was accomplished via the coordination reaction between 2-methylimidazole and Co^2+^. The as-obtained BSCF-ZIF67 composite was subjected for pyrolysis at 750 °C in a N_2_ flowing atmosphere. During this process, ZIF-67 was carbonized under the catalysis by both Co^2+^ in the MOF and BSCF with the formation of metal-containing nitrogen-doped carbon (Co–N_x_–C), which kept the nanocages like morphologic shape as that of ZIF-67, and some minor phases such as Co_4_N and CoO/Co_3_O_4_ homogenously immobilized inside the nanocages, which anchored on the surface of BSCF particles to form a core–shell like structure. Proper ratio of BSCF to ZIF-67 is key to obtain such morphologic composites; therefore, the concentrations of precursors (cobalt nitrate and 2-methyl imidazole) of ZIF-67 were fixed, while the concentration of BSCF was gradually varied. Excessive amount of BSCF during the synthesis was found to cause serious sintering of the catalyst and the collapse of nanocages structure of the C-ZIF-67.

**Fig. 1:**
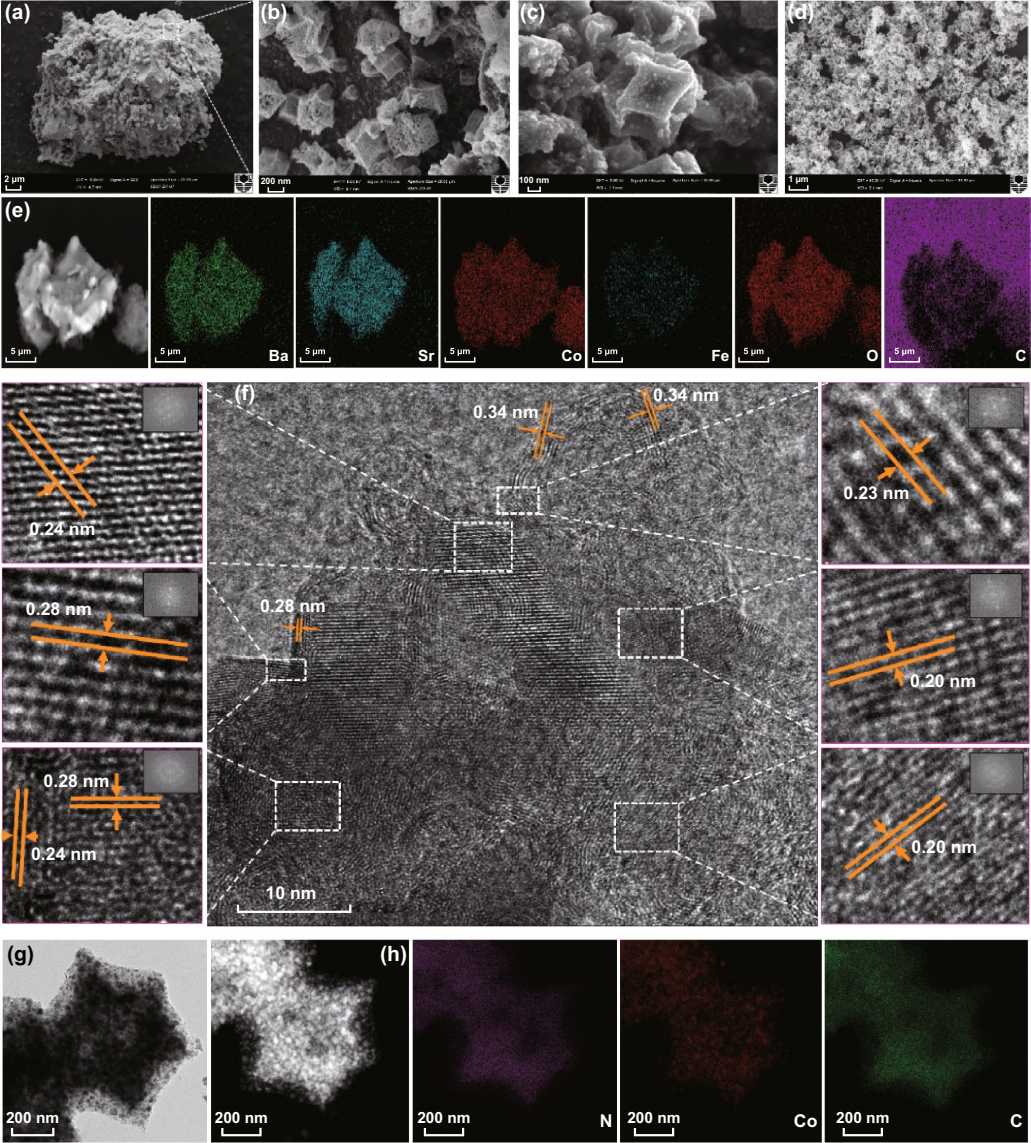
a, b Scanning electron microscopy (SEM) of BCZ1 sample illustrating the ZIF-67 crystals growth on BSCF particles, **c** magnified image of C-ZIF-67 crystals on BCZ1 and **d** BCZ2, **e** Corresponding energy dispersive X-ray spectroscopy (EDX) element mapping of BCZ2, **f** HRTEM of selected area of BCZ2, insets show the fast Fourier transform (FFT) patterns corresponding to six spots, **g** TEM image of carbonized ZIF-67 crystals, **h** STEM–HAADF image and corresponding elemental mapping of N, Co, and C elements of carbonized ZIF-67 crystals

It is well known that ZIF-67 takes a cubical morphology. It was noted that the C-ZIF-67 retained their typical shape of rhombic dodecahedron crystals without the appearance of agglomeration even after the pyrolysis, as confirmed by morphological analysis of the obtained composite materials using SEM (Fig. 1a–c). However, as compared to ZIF-67, the C-ZIF-67 nanocrystals experienced shrinkage and their surface had turned rough and porous (Fig. 1c, d), which should be the outcome of releasing of gases during the carbonization reaction. As shown in Fig. 1d, when the loading of BSCF was increased (such as BCZ2), multiple ZIF nanocubes had in situ grown onto the surface of micrometer-sized particles and enveloped the BSCF substrate tightly to form a core–shell-like structure. According to the EDX dispersive X-ray spectroscopy (EDS) mapping results (Fig. 1e), such micrometer-sized core should be the BSCF and the nanocubes covering the surface of core should be the C-ZIF-67. The immobilization of BSCF particles within well-interconnected porous nanocages may bring about several benefits. However, when the loading of BSCF is excessive (e.g. BCZ4), C-ZIF-67 nanocrystals and BSCF particles underwent serious sintering during pyrolysis, and composite materials with much larger size were developed, as a result of migration and agglomeration (Fig. S1).

HRTEM image of BCZ2 (Fig. 1f) reveals two well-resolved lattice fringes with an interplanar spacing of 0.23 and 0.20 nm, corresponding to the (111) and (220) lattice planes of metallic Co and Co_4_N, respectively. It may be figured out that cobalt in the ZIF-67 was converted into Co and Co_4_N nanoparticles after the pyrolysis, which were strongly and evenly implanted in the porous polyhedron of C-ZIF-67. Meanwhile, neighbouring interplanar spacing of 0.34 nm in the outer shell, corresponding to the (002) layer of graphitic carbon, confirms the formation of crystalline/graphitic carbon. Such carbon would obviously promote the electronic conductivity. Thus, it may be evaluated that ZIF-67-derived outer shell consisting of Co–N-doped graphitic carbon is highly active for both ORR and OER. HRTEM image (Fig. S2) further reveals that such Co-based nanoparticles were wrapped by multi-walled carbon layers, which suggests that Co nanoparticles may foster the graphitization of carbon during the carbonization [21, 22], and Co species are protected against aggregation/leaching during repetitive cycling. On the other hand, lattice spacing of 0.24 and 0.28 nm attributing to two major distinct crystal planes of (111) and (110), respectively, for BSCF perovskite, reveals the presence of BSCF perovskite structure, which are well consistent with the inset fast Fourier transform (FFT) patterns. It is also clearly visible from the TEM images that C-ZIF-67 inherited the original structure of its precursor after the pyrolysis (Fig. 1g). Meanwhile, Co/Co_4_N species were thoroughly anchored in the framework of carbon, which may be visualized in the corresponding EDX mapping that shows the uniform distribution of elements N, Co and C in the whole architecture (Fig. 1h).

**Fig. 2:**
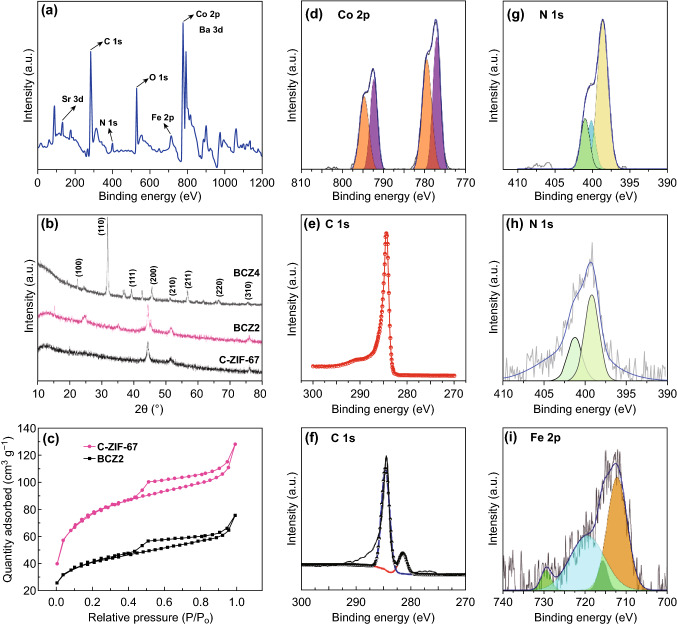
**a** XPS survey spectrum of BCZ2 composite. **b** XRD patterns of carbonized ZIF-67, BCZ2 and BCZ4 samples. **c** N_2_ adsorption/desorption isotherms of carbonized ZIF-67 and BCZ2 composite. **d** High-resolution deconvoluted XPS spectrum of Co 2*p* of BCZ2, High-resolution deconvoluted XPS spectrum of C 1*s* of **e** C-ZIF-67 and **f** BCZ2 composite. High-resolution deconvoluted XPS spectrum of N 1*s* of **g** carbonized ZIF-67 **h** BCZ2 composite. **i** High-resolution deconvoluted XPS spectrum of Fe 2*p* of BCZ2

Ex situ XRD characterization was employed to understand the phase evolution of C-ZIF-67, BCZ2 and BCZ4 during the carbonization process (Fig. 2b). Obviously, the distinct maxima at 2*θ* of ~ 44°, 52° and 75.8° correspond well to the Co (111), Co (200), and Co_4_N (220) reflections [23–25], demonstrating the effective transformation of ZIF-67 to metallic Co and Co_4_N after the pyrolysis in nitrogen atmosphere at 750 °C. Furthermore, BCZ2 also replicated the similar trend as those of C-ZIF. Hence, it confirms the successful growth of ZIF-67 crystals on BSCF surface, which is also witnessed by SEM (Fig. 2b) and TEM (Fig. 2g) micrographs. Interestingly, in BCZ2, an additional peak emerged at 2*θ* ~ 25.9°, attributing to partially graphitic carbon, which is in line with HRTEM of BCZ2. It indicates that amorphous phase of carbon had transformed into crystalline one, which is an added advantage. Actually, it occurred because Co catalysed the degree of graphitization of carbon at higher temperature (≥ 700 °C) [26]. Clearly, when the loading of BSCF was further increased (BCZ4), cubic phase of BSCF becomes more obvious; nevertheless, at higher loading, BSCF particles underwent agglomerations as illustrated by SEM (Fig. S1).

Thermal analysis of ZIF-67, BCZ1, BCZ2 and BCZ4 composites was conducted by TGA in air, presented the total carbon content of 64.2, 56.2, 47.2 and 36.5%, respectively (Fig. S3). BET textural properties were evaluated by nitrogen adsorption/desorption isotherms. BET curves of BCZ2 and ZIF-67 are illustrated in Fig. 2c, and the values are listed in Table S1. BCZ2 demonstrated type-IV isotherm with a typical H1 hysteresis loop, which corresponds to the hierarchical mesoporous structure. BET surface area of BCZ2 was found to be 128.2 m^2^ g^−1^, demonstrating that the composite material had large surface area even after the calcination and the incorporation of BSCF. Such high surface area should be mainly attributed to the porous nanocages that deposited on the BSCF surface. The large surface area would be conducive to expose ample active sites, speedup the mass transfer kinetics (electrolyte/oxygen) and subsequently contribute to enhance the OER/ORR [27, 28]. However, when the loading of BSCF was increased (BCZ4), BSCF particles underwent sintering and then led the carbon nanocages to experience collapse, leading to the agglomeration of the composite material.

XPS was conducted to further elaborate the formation and transformation of binding states of Co, N, C and Fe during the synthesis. Survey spectra of BCZ2 are displayed in Fig. 2a, which represents that the representative peaks of C-ZIF-67 and BSCF are concomitantly present in the composite. The deconvoluted high-resolution Co 2p XPS spectra of BCZ2 meticulously demonstrate the desired features in the BCZ2 composite (Fig. 2d). The binding energies in Co 2p corresponding to 778.2 and 793.1 eV are assigned to metallic Co (Co^0^), which contributes to ORR activity, whereas the peak at ~ 781.0 eV is attributed to Co_4_N [23, 25, 29]. On the other hand, the binding energy of 779.7–781.5 eV is attributed to CoO (2*p*_3/2_) and Co_3_O_4_ (2*p*_3/2_) [30], while the peak at 795.90 eV is ascribed to CoO (2*p*_1/2_) and Co_3_O_4_ (2*p*_1/2_). Thus, it may be figured out that Co^2+^, Co^3+^ peaks and Co_4_N species may overlap in the 779.7–781 eV region, which are known to enhance OER activity [25, 31, 32]. High-resolution C 1 s spectrum of C-ZIF-67 demonstrates a single peak at 285.1 eV, attributing to the existence of N-doped graphitic carbon (C–N), a major contributor to ORR activity in C-ZIF-67 (Fig. 2e). This bond occurred because N is capable of bonding with three carbon atoms and its compatibility with *sp*^2^ carbon network is higher, leading to the bulk defects in graphitic C network [33]. The same peak as that was in C-ZIF-67 was also transcribed in BCZ2 composite (Fig. 2f) accompanied by a new prominent peak at 281.4 eV. The new peak has resulted from Fe_3_C [34, 35], which promotes the ORR activity [36]. The high-resolution N1*s* peaks of carbonized ZIF-67 (Fig. 2g) and BCZ2 (Fig. 2h) were deconvoluted into predominantly three forms of N functional groups: pyridinic N species (398.4 eV), graphitic N species (400.7 eV) and Co–N_x_ (399.3 eV) moieties [37]. The pyridinic and graphitic N species in the catalyst favour the electrocatalytic activity. Actually, pyridinic N has the ability of accepting electrons from C atoms present in the vicinity, whereas graphitic N promotes the transfer of electrons. The optimal catalyst (BCZ2) had the sufficient amount of pyridinic and graphitic N species, which would contribute to enhance ORR. The high-resolution spectra of N1s also reaffirm the presence of covalent bond between Co and pyrrolic N species, involving the electron transfer from Co to N [38]. After the in situ growth of ZIF-67 on BSCF, a new peak emerged, centred at about 399.1 eV, that may be designated as Fe-coordinated N moieties (Fe-N_x_) [39]. Fe*-*N_x_ species are considered as the contributor to ORR [39, 40]. Consequently, it may be figured out that N present in C-ZIF-67 serves as the anchoring points between BSCF and C-ZIF-67 [33]. Meanwhile, the high-resolution Fe 2*p* spectrum of BCZ2 (Fig. 2i) can be deconvoluted into two major signals: the first peak may correspond to the overlapping of Fe-coordinated N, Fe-N_x_ configuration (712.9 eV) [40], and 2*p*_3/2_ orbitals of Fe^2+^ (710.2 eV) and Fe^3+^ (714.0 eV) species [24, 41], while the later peak positioned at 720.1 eV may be attributed to the zero valence state of Fe. On the other hand, the minor peaks located around 716.8 and 728.1 eV could be designated to the satellite peaks. The concomitance of Fe_2_O_3_ and Fe_3_C bestowed the bi-functional properties because Fe_2_O_3_ modulates the electronic structure of Fe_3_C, and Fe_3_C in turn promotes the electron mobility of Fe_2_O_3_ [39].

### Electrochemical Characterizations of Catalysts

**Fig. 3:**
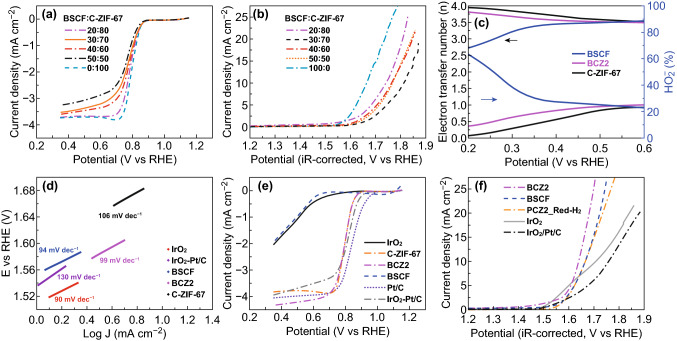
**a** ORR and **b** OER polarization curves of composite samples prepared by physical mixing using different ratio in an oxygen-saturated 0.1 M KOH at a rotating speed of 1600 rpm. **c** Electron transfer number and percentage of HO_2_^−^ of BSCF, C-ZIF-67 and BCZ2 composites at various potentials. **d** Tafel plots, **e** ORR and **f** OER polarization curves of various samples including BCZ2 composite in an oxygen-saturated 0.1 M KOH at a rotating speed of 1600 rpm

To correlate the physicochemical properties/structural properties and catalytic performance of the catalysts, the electrocatalytic activity was evaluated using three-electrode system in O_2_-saturated KOH solution (0.1 M). We first tested the electrocatalytic activity based on ORR and OER of BSCF and carbonized ZIF-67. Figure 3 illustrates the linear sweep voltammograms (LSV) of the electrodes. BSCF generally shows good OER activity, which is in agreement with the literature [11, 42], while its ORR activity is poor. On the other hand, carbonized ZIF-67 shows favourable ORR activity, whereas its OER activity is worse than BSCF. As the formation of a composite from an OER electrocatalyst and an ORR electrocatalyst is the general way to get a bi-functional catalyst. Therefore, the composites of perovskites and Co–N_x_–C were prepared by physical mixing through ultrasonication process. For the purpose of comparison, we synthesized various BSCF and C-ZIF-67 composites of different mass ratios based on physical mixing of pre-formed BSCF and C-ZIF-67, to confirm the superiority of current in situ growing method for the synthesis of the nano-micro-composites. Upon the investigation of OER/ORR performance via RDE at the scan rate of 5 mV s^−1^ in O_2_-saturated 0.1 M KOH solution (Fig. 3a, b), it was revealed that the physically mixed composites demonstrated some bi-functional activity, despite that the OER and ORR activity of these physically mixed composite materials was much inferior to the individual OER activity of BSCF and ORR activity of C-ZIF-67, respectively. Such reduction in catalytic activity of the composite materials for ORR and OER may be the outcome of blocking of active sites of BSCF and C-ZIF-67, or the lack of intimate contact between them.

Consequently, the limited activity of physically mixed composite material motivated us in situ growing of ZIF-67 nanocrystals on the surface of BSCF. This effect may impart intimate contact between BSCF and ZIF-67, leading to the suppression of agglomeration of BSCF particles and inhibition of leaching of Co species from N-doped carbon network. In turn, it may lead to the modulation in electronic structure and overcome the interfacial resistance to bring synergistic effect. The ORR and OER activities of different composites (BCZ1, BCZ2, BCZ3 and BCZ4) were comparatively investigated by RDE (Fig. S4), and BCZ2 composite was found as an optimal catalyst based on ORR and OER performance. Subsequently, ORR performance of optimal composite (BCZ2) and benchmark precious metal-based catalysts was studied (Fig. 3e). It was found that BCZ2 and C-ZIF-67 displayed exactly analogous onset potential (*E*_onset_ ≈ 0.45 V) and half-wave potential (*E*_1/2_ ≈ 1.56 V). Interestingly, BCZ2 composite material had the highest diffusion limiting current density, even higher than the benchmark Pt/C catalyst and IrO_2_-Pt/C catalysts at 5 mV s^−1^ scan rate and 1600 rpm, which implies that a synergistic effect between BSCF and C-ZIF-67 was likely established in the composite that further improved the ORR. In contrast, pristine BSCF was found to have poor ORR activity. Interestingly, it is also visualized in Fig. S4b that ORR activity (onset potential, half-wave potential and limiting current density) of catalysts increased with the increase in the loading of BSCF in the composite. It suggests that BSCF also likely became active for ORR owing to the development of new phases at the interface of BSCF and C-ZIF-67, as suggested by XPS.

To gain the deeper insight into the ORR kinetics of BCZ2, RRDE technique was employed to monitor the overall ORR pathways. Figure S5 displays the disk current (*I*_d_) and the ring current (*I*_r_, multiplied by 10) using the BCZ2 catalyst at several rotation speeds. It can be observed that the values of current rise as the rotation rates increase, whereas onset potential was found constant at different rotation speeds in the potential range of 0.2–0.5 V, which depicts the linearity with same slopes. Conspicuously, the *I*_r_ was far less (in spite of 10-fold multiplication) as compared to *I*_d_, pointing out that a little amount of HO_2_^−^ was generated in the course of ORR activity and preferably leading to 4*e*^−^ pathway. The average H_2_O_2_ yield and average number of transferred electrons of BCZ2, BSCF and C-ZIF electrocatalysts were computed using RRDE (Fig. 3c). The average H_2_O_2_ yield of BCZ2 catalyst was around 18% in the whole test potential range, referring a high catalytic activity towards ORR. The corresponding average number of transferred electrons per O_2_ (*n*) was approaching 4, implying that 4*e*^−^ pathway is followed by BCZ2 for ORR activity, which is known for complete reduction of O_2_. It is worth noting that BSCF delivered several pathways over the whole potential range as witnessed by different electron transfer numbers and peroxide (HO_2_^−^) generation rate. On the other hand, BCZ2 followed a single path (i.e. four-electron route) which implies that C-ZIF in the composite has promoted towards the 4e^−^ pathway. In brief, ORR indicators in terms of the electron transfer number as well as the amount of HO_2_^−^ generation of composite BCZ2 were close to the benchmark Pt/C.

OER performance of BCZ2, BCZ4, ZIF-67, BSCF, IrO_2_ and IrO_2_ + Pt/C was also interrogated via LSV polarization curves at the scan rate of 5 mV s^−1^ in O_2_-saturated alkaline solution (0.1 M KOH) in Fig. 3f. For comparison of different samples, the operating potential at current density of 10 mA cm^−2^ (*E*_*j*=10_) was employed. In this perspective, BCZ2 delivered a lowest potential of 1.64 V at 10 mA cm^−2^. Moreover, BCZ2 also exhibited an onset potential (1.52 V) comparable to that state-of-the-art IrO_2_ catalyst (Fig. 3f). In contrast, IrO_2_ and IrO_2_ + Pt/C exhibited higher potential on the same current density. Interestingly, the potential of BCZ2 was even 20 mV smaller than that of BSCF (1.66 V). Furthermore, the curve obtained by BCZ2 was much steeper, which signifies the enhanced electronic conductivity of composite material. The improved OER activity of BCZ2 may be associated with the combined effect of BSCF and CoN_4_, which can offer additional intrinsic OER active sites [24]. To figure out the influence of BSCF perovskite oxide in BCZ2 composite, it was reduced in the H_2_ atmosphere. As a result, a substantial decline in the OER activity was observed which testifies the significant contribution of BSCF in OER performance. Moreover, the integration of BSCF into N-doped carbon porous network can efficiently modulate the electronic structure, BSCF being *p*-type semiconducting material establishes a *p*–*n* junction with N-doped carbon (*n*-type) and fine-tune the electron density [9]. Subsequently, we evaluated the Tafel plots to examine the intrinsic catalytic OER kinetics (Fig. 3d). Clearly, the obtained Tafel slope of BCZ2 (97 mV per decade) was comparable to BSCF and IrO_2_ and smaller than IrO_2_ + Pt/C (130 mV per decade), pointing out that BCZ2 demonstrated excellent OER kinetics as a result of combined effect of BSCF, CoN_4_ and the development of new phases at the interface. Thus, the Tafel slopes are in good agreement with the OER activity profiles obtained through LSV curves.

The overall bi-functional activity of oxygen electrode may be quantified by the activity parameter Δ*E,* the difference between Ej10 (OER) and E1/2 (ORR). For the optimal BCZ2 composite electrode, Δ*E* was found to be 0.83 V, which is even better than the bimetallic IrO_2_ + Pt/C (1:1) catalyst (0.88 V) constructed from the benchmark electrocatalysts of IrO_2_ for OER and Pt/C for ORR (Fig. 3a). The low Δ*E* value of BCZ2 corroborates that it is promising to apply BCZ2 as air electrode for rechargeable metal air batteries.

### Zn–air Battery Testing of Catalysts

**Fig. 4:**
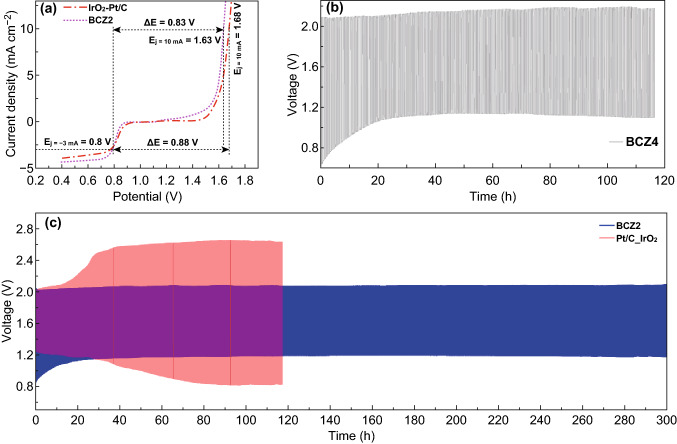
**a** Overall polarization curves of BCZ2 and commercial Pt/C and IrO_2_ in 0.1 M KOH. Rechargeable zinc–air battery tests. **b** galvanostatic charge/discharge test based on BCZ4and **c** BCZ2 and Pt/C and IrO_2_ mixture catalysts tested for Cyclic stability at 5 mA cm^−2^ for 300 h

As a proof of concept, rechargeable ZABs were assembled to practically implement the bi-functional activity and to evaluate the long-term cyclic stability of BCZ2, BCZ4, and Pt/C + IrO_2_ catalysts under similar conditions. Overall, it can be observed that the ZAB with the BCZ2 air electrode displayed remarkably stable charging/discharging cycles for 300 h without any appreciable decline in the performance (Fig. 3c). On the other hand, the charging/discharging performance with the Pt/C + IrO_2_ air electrode dropped even after few hours of operation. In brief, the BCZ2 air electrode exhibited excellent OER (charging) and ORR (discharging) performance corresponding to the potential gap of 0.83 V. In contrast, OER/ORR performance of Pt/C + IrO_2_ air electrode started to decline after 30 h, corresponding to the voltage gap of 1.31 V and voltaic efficiency of 47%, while charging/discharging potential gap was 1.81 V (31% voltaic efficiency) after 100 h. Interestingly, the voltage gap of BCZ2 was consistent without any obvious change in the activity even after 1800 cycles for 300 h. Thus, the results further reveal the excellent rechargeability and durability of BCZ2 air electrode for ZAB. For comparison, by using BCZ4 as the air electrode (Fig. 4b), it was found that BCZ4 had a larger potential gap (1.11 V) and lower voltaic efficiency (51%). Consequently, it emphasizes that optimal weight ratio of BSCF to C-ZIF-67 is vital to achieve the bi-functionality and cycling stability in the in situ derived nano-micro-composite; thus, BCZ2 was found superior to BCZ4 in terms of both activity and stability. To ensure the high activity and stability of in situ grown ZIF polyhedrons on BSCF particles (BCZ2), physical mixtures of BSCF and C-ZIF-67 catalysts in different ratios (Fig. S6) were tested by Zn–air batteries. It was revealed that BCZ2 was far more stable than the physically mixed catalysts because charging/discharging cyclic stability in physically mixed catalysts declined within the less than half of duration of 300 h, leading to the higher potential gaps. Likewise, BCZ2 demonstrated superior activity and stability as compared to its individual constituents BSCF (Fig. S7) and C-ZIF-67 (Fig. S8).

**Fig. 5:**
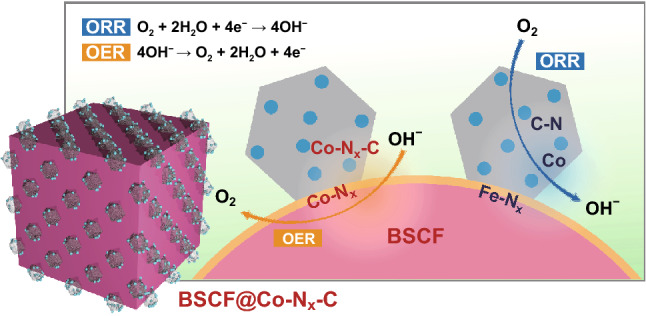
Possible mechanism of OER and ORR and for synergy between C-ZIF-67 and BSCF composite

The outstanding bi-functional catalytic activity of BCZ2 is likely the outcome of synergistic effect between BSCF and C-ZIF-67 in the composite. The remarkable OER performance of BCZ2 may be associated with the simultaneous installation of BSCF and Co_4_N in the composite. Shao-Horn group, using molecular orbital principles, established that BSCF has surface Co cation *e*_g_ filling, *e*_g_ = 1, which may impart their reasonable binding with oxygen (i.e. neither weak, *e*_g_ > 1, nor strong, *e*_g_ < 1) in alkaline media [43], thus leading to the significantly high OER activity [11]. On the other hand, the excellent ORR activity may be endowed with N-doped carbon. N doping in the ZIF-67 renders the composite material more electron surplus by electron pair in pyridinic N and breaking the electro-neutrality of carbon present in the vicinity [44], thus featuring high ORR activity and stability [45–50]. Subsequently, N-doped carbon delivers electrons to π* orbital of adsorbed O_2_ gas and activating it, resulting in promoting the ORR activity. Meanwhile, the donation of electrons to BSCF also upgraded the covalence between lattice oxygen and Co, eventually rising the OER activity, which is an added advantage [51]. That’s the reason, OER activity of BCZ2 surpassed the OER activity of BSCF. As the excellent oxygen adsorption behaviour and ORR activity of N-doped carbon are comparable to Pt [52]. Therefore, the spillover effect (as dominant in Pt/metal oxide) may govern the synergistic effect for the bi-functional catalytic performance of BSCF/C-ZIF-67 composite. In case of ORR, OH^−^ produced on N-doped carbon in C-ZIF-67 may spillover onto the BSCF surface by virtue of their priority to adsorb on metal oxides. In turn, this effect could yield more N-doped carbon active sites for ORR and promoting the ORR activity. In the similar fashion, O_2_ generated in OER process on BSCF surface spills over on the N-doped carbon and upgrading the OER activity. Moreover, the electronic cloud extending from the N-doped carbon framework (C-ZIF-67) to BSCF [51], leading to the formation of Co–N_x_ and Fe–N_x_ species at the interface (Fig. 5). In turn, these intermediate species not only anchored the BSCF and ZIF-67 crystals together but also contributed well to the electrocatalytic performance. Thus, it may be figured out that N present in ZIF-67 serves as the anchoring points between BSCF and ZIF-67. These anchoring sites strongly immobilized the BSCF particles inside the N-doped porous carbon network and protected them against sintering/agglomeration. It is also obvious in case of higher loading of BSCF (BCZ4, Fig. S1), wherein N could not provide sufficient anchoring sites leading to the agglomeration of BSCF particles.

In brief, in situ growth of ZIF-67 crystals onto BSCF particles presented superior bi-functional activity and outstanding stability. These excellent performances may be associated with unique architecture, which provided following lucrative features: (1) offering the resistance against sintering/aggregation of BSCF particles during repetitive charging/discharging cycles, (2) providing intimate contact between BSCF/metal species and graphitized carbon to facilitate quick electron charge transfer, (3) exposing plentiful active sites for electrochemical reactions, (4) providing sufficient surface area and diffusion channels for the efficient diffusion of oxygen gas for ORR/OER [27, 28, 53, 54], (5) encapsulating Co species by carbon layers, which induced the higher degree of graphitization to carbon, providing excellent conductivity throughout the electrocatalyst and avoiding the employment of additional conductive additive and (6) suppressing the detachment of ZIF-67 crystals from BSCF by establishing the electrostatic attraction due to electronic cloud between mesoporous N-doped carbon framework and BSCF and promoting the structural stability.

## Conclusion

A novel high-performance bi-functional electrocatalyst was designed, wherein BSCF perovskite particles were immobilized inside polyhedrons by in situ growth of ZIF-67 nanocrystals (BSCF@ZIF-67). Following the pyrolysis, BSCF particles were found encapsulated inside the Co–N_x_–C nano cages (BSCF@ Co–N_x_–C). N-doped porous carbon network endowed the composite material several benefits: anchoring the BSCF particles and protected against aggregation, providing high surface area to expose abundant active sites and bridging between BSCF particles and Co–N_x_–C to ensure efficient charge transfer, unravelling the high intrinsic activity of BSCF perovskites. Moreover, plenty of Co-based nanoparticles were evenly embedded in N-doped carbon framework and thus supplemented the OER/ORR activity. On the other hand, N served as anchoring sites and gave birth to Fe–N_x_ and Co–N_x_ species at the interface, chemically fastened the BSCF particles and N-doped carbon intimately and avoiding the leaching of metallic species into alkaline solution, which obviously translated into improved ORR/OER performance. Consequently, the composite material demonstrated the superior reaction kinetics and it outperformed their individual constituents in terms of OER as well as ORR in Zn–air batteries. Thus, the unique architecture while upgrading the surface features enabled the composite electrocatalyst as a cathode to sustain over long-term cycling. This study may usher new avenue to design cost-effective composite materials based on MOFs and perovskites and exploiting their mutual benefits in electrocatalysis and other catalytic systems.

## Electronic Supplementary Material

Below is the link to the electronic supplementary material.

Supplementary material 1 (PDF 632 kb)
