# Supplementary material for: A Porous Nano-Micro-Composite as a High-Performance Bi-Functional Air Electrode with Remarkable Stability for Rechargeable Zinc–Air Batteries
Source: Nanomicro Lett. 2020 Jun 17;12:130. doi: 10.1007/s40820-020-00468-4 (PMC7770687; doi:10.1007/s40820-020-00468-4)
Supplement: Supplementary file 1 — Supplementary material 1 (PDF 632 kb) [file 40820_2020_468_MOESM1_ESM.pdf]

Supporting Information for

A Porous Nano-Micro Composite as a High-Performance Bi-Functional Air Electrode with Remarkable Stability for Rechargeable Zinc-Air Batteries

Yasir Arafat[1], Muhammad Rizwan Azhar [1, 2], Yijun Zhong[1], Xiaomin Xu[1], Moses O. Tadé[1], Zongping Shao[1,3,] *

[1]WA School of Mines: Minerals, Energy and Chemical Engineering (WASM-MECE), Curtin University, Perth, WA 6845, Australia

[2]School of Engineering, Edith Cowan University, Perth, WA 6027, Australia

[3]State Key Laboratory of Materials-Oriented Chemical Engineering, College of Chemical Engineering, Nanjing Tech University, Nanjing, Jiangsu 210009, People's Republic of China

*Corresponding author. E-mail: zongping.shao@curtin.edu.au (Zongping Shao)

Supplementary data

Table S1 BET surface area of pristine BSCF and carbonized composite materials

Electrocatalysts	BET surface area ($m^2\ g^{-1}$)
Pristine BSCF	0.184
BCZ1	132.90
BCZ2	128.21
BCZ3	104.13
BCZ4	86.42

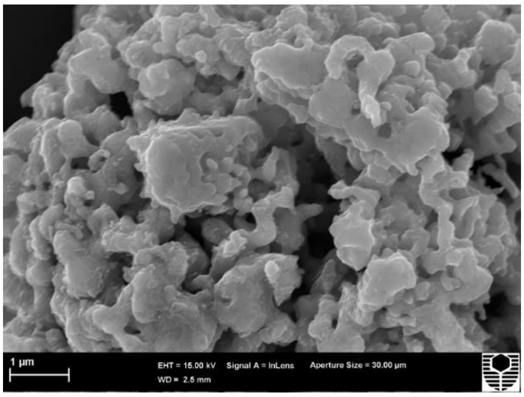

Fig. S1 SEM image of BCZ4

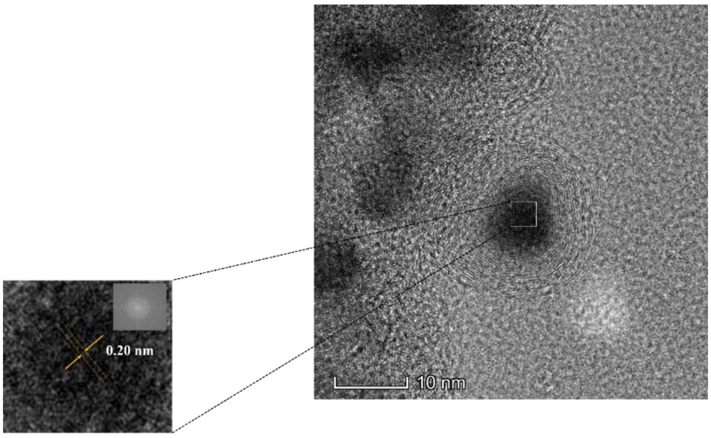

Fig. S2 HRTEM of selected area of BCZ2, insets show the fast Fourier transform (FFT) patterns corresponding to selected spot

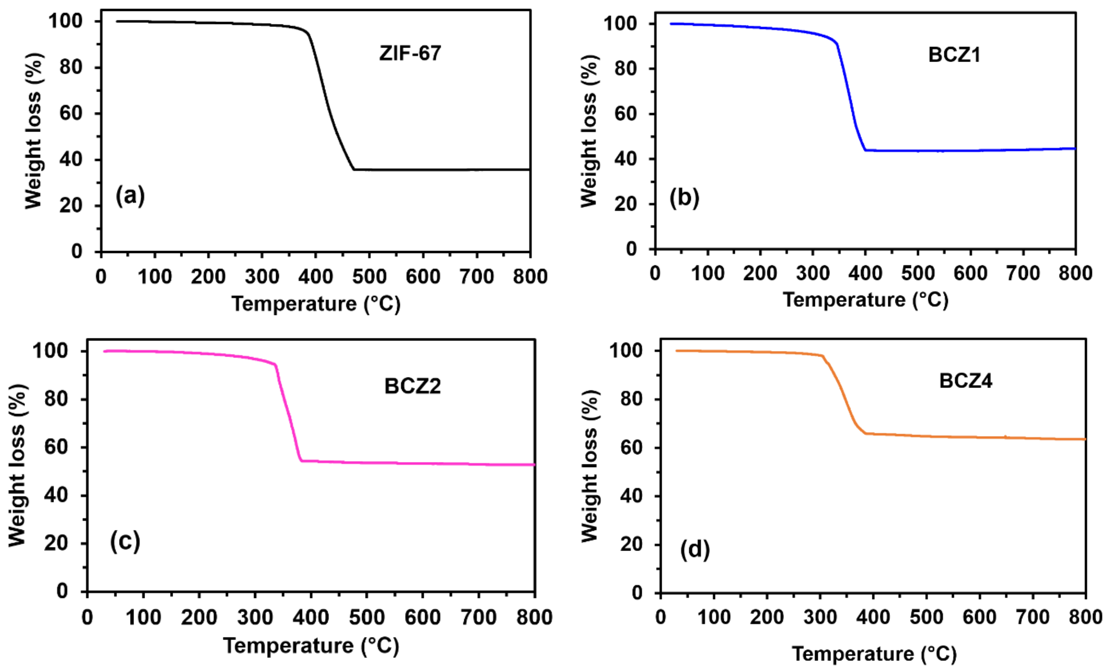

Fig. S3 TGA of (**a**) ZIF-67, (**b**)BCZ1, (**c**) BCZ2, and (**d**) BCZ4

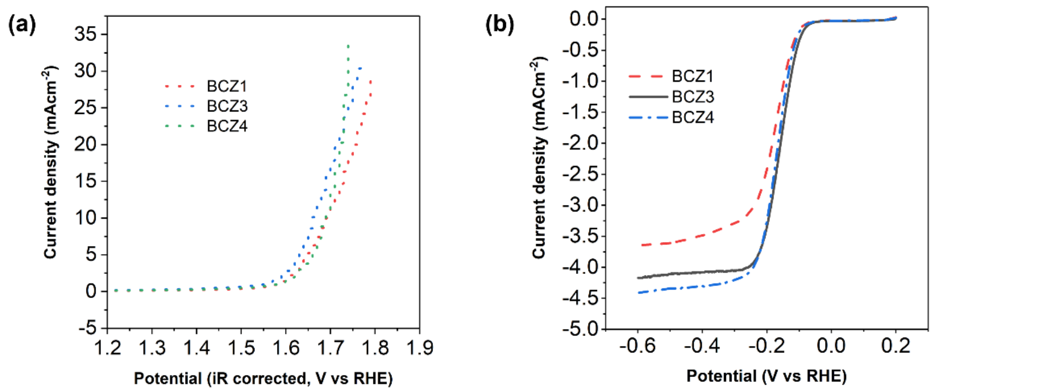

Fig. S4 (**a**) OER and (**b**) ORR polarization curves of various samples including BCZ1, BCZ3 and BCZ4 composites, prepared by in situ growth of ZIF-67 crystals on BSCF in an oxygen-saturated 0.1 M KOH at a rotating speed of 1600 rpm

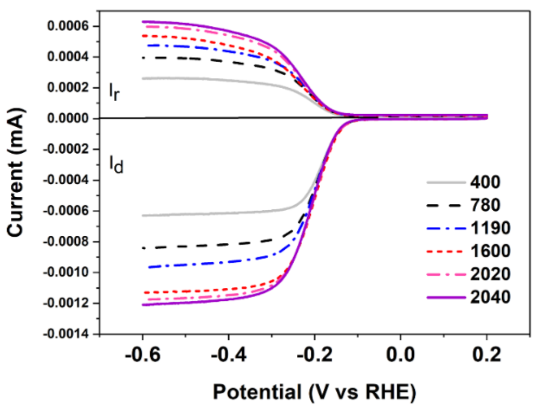

Fig. S5 RRDE test for BCZ2 composite

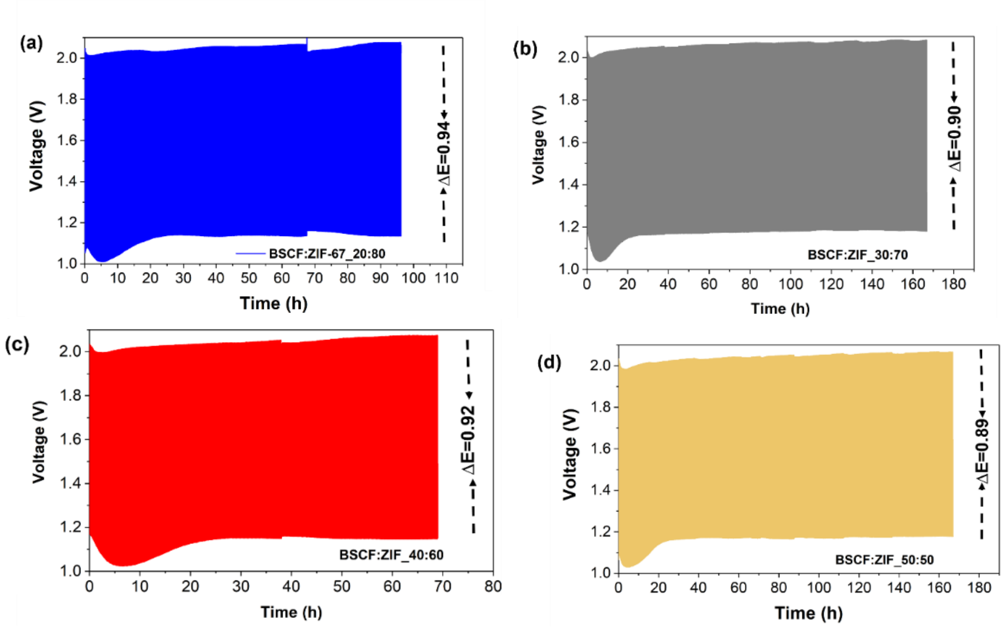

Fig. S6 Galvanostatic charge/discharge test based on physical mixing of BSCF and C-ZIF-67 in different ratios (**a**) BSCF:C-ZIF-67_20:80 (**b**) BSCF:C-ZIF-67 _30:70 (**c**) BSCF:C-ZIF-67 _40:60 and (**d**) BSCF:C-ZIF-67_50:50 mixture catalysts tested for Cyclic stability at 5 mA cm^{-2}

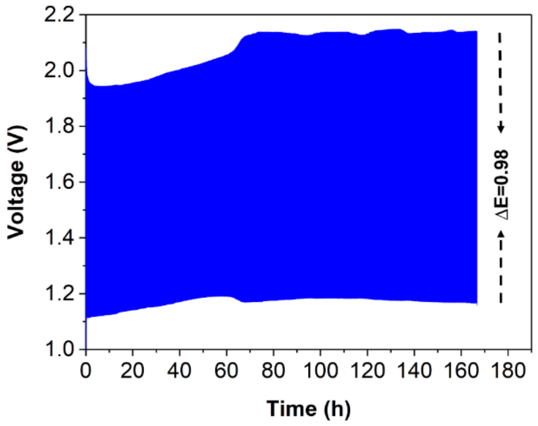

Fig. S7 Galvanostatic charge/discharge test based on BSCF catalyst tested for cyclic stability at 5 mA cm^{-2}

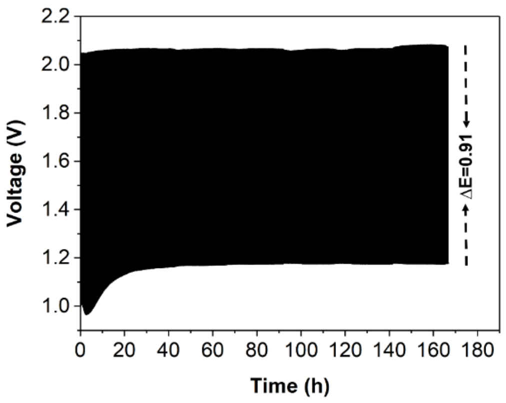

Fig. S8 Galvanostatic charge/discharge test based on C-ZIF-67 catalyst tested for cyclic stability at 5 mA cm^{-2}
